# Vertebral body tethering for idiopathic scoliosis: a systematic review and meta-analysis

**DOI:** 10.1007/s43390-023-00723-9

**Published:** 2023-07-11

**Authors:** Megan J. Roser, Geoffrey N. Askin, Robert D. Labrom, Syeda Farah Zahir, Maree Izatt, J. Paige Little

**Affiliations:** 1 Orthopaedics Department, Queensland Children’s Hospital, South Brisbane, Australia; 2 Biomechanics and Spine Research Group (BSRG), Centre for Children’s Health Research, Queensland University of Technology (QUT), Brisbane, Australia; 3 Queensland Cyber Infrastructure Foundation (QCIF), Facility for Advanced Bioinformatics, The University of Queensland (UQ), Brisbane, Australia

**Keywords:** Vertebral body tethering, Adolescent idiopathic scoliosis, Systematic review, Orthopaedics, Surgery

## Abstract

**Purpose:**

Vertebral body tethering (VBT) is a recent procedure to correct and reduce spinal curves in skeletally immature patients with adolescent idiopathic scoliosis (AIS). The purpose of this systematic review and meta-analysis is to determine the expected curve reduction and potential complications for adolescent patients after VBT.

**Methods:**

PubMed, Embase, Google Scholar and Cochrane databases were searched until February 2022. Records were screened against pre-defined inclusion and exclusion criteria. Data sources were prospective and retrospective studies. Demographics, mean differences in Cobb angle, surgical details and complication rates were recorded. Meta-analysis was conducted using a random-effects model.

**Results:**

This systematic review includes 19 studies, and the meta-analysis includes 16 of these. VBT displayed a statistically significant reduction in Cobb angle from pre-operative to final (minimum 2 years) measurements. The initial mean Cobb angle was 47.8° (CI 95% 42.9–52.7°) and decreased to 22.2° (CI 95% 19.9–24.5°). The mean difference is − 25.8° (CI 95% − 28.9–22.7) (*p* < 0.01). The overall complication rate was 23% (CI 95% 14.4–31.6%), the most common complication was tether breakage 21.9% (CI 95% 10.6–33.1%). The spinal fusion rate was 7.2% (CI 95% 2.3–12.1%).

**Conclusion:**

VBT results in a significant reduction of AIS at 2 years of follow-up. Overall complication rate was relatively high although the consequences of the complications are unknown. Further research is required to explore the reasons behind the complication rate and determine the optimal timing for the procedure. VBT remains a promising new procedure that is effective at reducing scoliotic curves and preventing spinal fusion in the majority of patients.

**Level of evidence:**

Systematic review of Therapeutic Studies with evidence level II–IV.

**Supplementary Information:**

The online version contains supplementary material available at 10.1007/s43390-023-00723-9.

## Introduction

AIS is a three-dimensional spinal deformity demonstrating a lateral curve with a Cobb angle of > 10°, and exclusion of an underlying cause [1, 2]. AIS has an onset at puberty and female predominance [1–3]. AIS is painless in adolescence, but due to the progressive nature can cause issues with pain, cardiopulmonary function, cosmesis and early death in adulthood [4]. AIS is known to progress during the pubertal growth spurt, the risk of progression can be predicted using the Cobb angle, skeletal maturity and menarche status [5].

Current treatments are determined based on the major curve Cobb angle and the bone age. In skeletally immature patients, a major curve Cobb angle of < 25° can be managed conservatively with serial radiographs to assess progression [6]. A major curve Cobb angle 25–45° is expected to progress and is often managed with bracing [7]. A major curve ≥ 45° is likely to progress to cause disability and is expected to need surgical correction. Skeletally mature patients with a Cobb angle of ≤ 45° are unlikely to need surgical intervention as their risk of progression has significantly reduced [6].

Posterior spinal instrumented fusion (PSIF) is the gold standard surgical procedure, which reduces the deformity and fuses the spine to prevent progression [8]. The concern for early PSIF is it will halt remaining spinal growth and reduce the thorax volume, decreasing pulmonary function [9].

Another surgical option is growing rods, which require surgery every 4–6 months to manually lengthen as the spine grows [10]. Disadvantages include multiple general anaesthetics, risk of wound infections, spontaneous auto-fusion and eventual PSIF [11–13].

Young, skeletally immature patients with severe curves provide a unique challenge for surgical intervention. Surgeons must weigh up the curve severity and risk of progression, against preserving remaining growth of the thorax and spinal height. These patients may have been unable to tolerate a brace, or their curves may have progressed beyond the upper limit for bracing, yet they remain skeletally immature and therefore at risk of further progression of their spine deformity.

VBT is an emerging surgical procedure intending to reduce the curve and prevent progression with one surgery, whilst preventing a PSIF. VBT secures a polyethylene tether to the convex surface of the major curve with screws at multiple vertebral levels [14]. It is thought that VBT induces asymmetrical growth in the spine via the Hueter–Volkman law [15]. VBT does not involve spinal fusion and therefore allows the continued spinal growth. Since the concept was introduced off-label in 2010 [16], and FDA approved in 2019, multiple international centres have performed the VBT procedure.

The authors are not aware of a meta-analysis that compares outcomes from VBT exclusively. This is a systematic review and meta-analysis of studies solely investigating VBT outcomes in AIS. The primary purpose of this study was to determine if VBT was successful at reducing the spinal curvature. The second purpose was to determine the complication rate, and how many patients ultimately required a spinal fusion.

## Materials and methods

### Study selection

A systematic review was performed according to the Preferred Reporting Items for Systematic Review and Meta-Analysis (PRISMA) guidelines [17]. Studies met the inclusion criteria if they (1) investigated the treatment of AIS, (2) in patients with a main thoracic curve, by (3) performing anterior VBT, (4) in skeletally immature patients, (5) reported the pre- and post-operative major curve Cobb angle and (6) had minimum 2 years of follow-up.

Studies met the exclusion criteria if they (1) solely investigated non-idiopathic scoliosis e.g. neuromuscular (2) performed finite element analysis, (3) used non-human models, was a 4) review article, (5) conference abstract, (6) case report, or (7) only detailed surgical technique.

PubMed, Google Scholar, Embase and Cochrane were searched and retrieved all VBT clinical studies published in English. The following keywords were used: ‘vertebral body tethering’ and ‘adolescent idiopathic scoliosis’. Medical Subject Heading (MeSH) vocabularies were selected when retrieving articles. Databases were searched using the advanced function on 17 February 2022.

### Data extraction

Two researchers (M.R. and J.P.L) screened all results against the inclusion and exclusion criteria. A list of studies from each source was printed and duplicates removed. Article abstracts were reviewed and discussed until a consensus was obtained. Full-text articles were retrieved, downloaded and manually organised, without automation tools. Citations were downloaded into EndNote.

A data extraction form was developed [18] and used by one researcher (M.R.). Corresponding authors of included studies were contacted for additional information when required.

### Data outcomes

To determine the clinical success of the VBT procedure, the main measured outcome is the Cobb angle of the major curvature of the spine [19]. This was recorded pre-operatively and at final follow-up, which was mostly 2 years, but for some studies was longer than 2 years. The mean major curve Cobb angle, range of values and standard deviation (SD) were recorded. Other measured outcomes are surgical details and surgical complications.

### Data synthesis

For the meta-analysis, a random-effects model was fitted using DerSimonian and Laird method (DL) for estimating heterogeneity [20]. Heterogeneity was examined using the Higgins I2 statistic [21]. I2 values of 25%, 50% and 75% indicated low, moderate and high heterogeneity. Forest and funnel plots were presented for visualisation of summary effect sizes and publication bias. All analyses were conducted using metafor package in R (version 4.0.4) (2021).

Given the homogeneity between the inclusion criteria of the studies, they were compared against each other for overall mean difference in major curve Cobb angle. In cases when there was no SD available, the study was excluded from the meta-analysis. Bar charts were created to visually display the mean change in major curve Cobb angle. The correlation coefficient was unable to be calculated. A conservative correlation coefficient was chosen using 0.4. The SD of the mean difference was calculated using the formula [22];

$${\text{SD}}_{{\text{E,change}}} = \sqrt {{\text{SD}}_{{\text{E,baseline}}}^{{2}} } + {\text{SD}}_{{\text{E,final}}}^{{2}} - \left( {2x\,{\text{Corr}} \,x \,{\text{SD}}_{{\text{E,baseline}}}\, x \,S{\text{D}}_{{\text{E,final}}} } \right)$$

Small study effects owing to potential publication bias, poor methodological quality, true heterogeneity or chance were analysed with contour-enhanced funnel plots. To assess for selective reporting biases, the methods and the results sections were compared, and clinical knowledge was applied to assess for any discrepancy. The National Heart, Lung and Blood Institute (NHLBI) quality assessment tool for pre–post studies with no control group [23] was used to assess for the risk of bias in the selected studies.

## Results

**Fig. 1:**
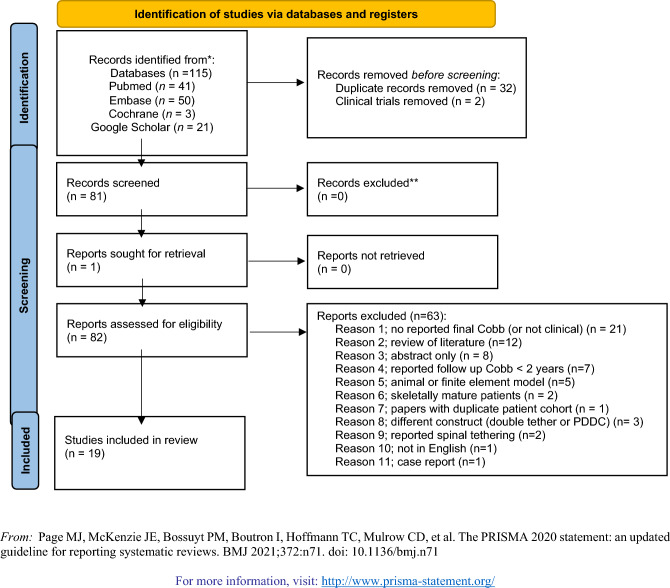
Flow chart for article selection.

115 studies were identified using the above search strategy. 19 studies with a total of 677 patients met the inclusion criteria. Figure 1 displays the PRISMA flowchart for article selection. 83 articles were assessed for eligibility, 63 were excluded and the reasons are stated in Fig. 1.

### Included studies

19 studies met the inclusion criteria (Table 1). 12 studies had a subset of patients that were excluded (appendix 1). 13 of the studies were rated as low, and 6 were rated as moderate risk of bias by the NHLBI criteria (appendix 2a and b).

**Table 1:** Study characteristics and patient demographic

Author	Country	Study	Year	Evidence level	Risk of bias	*n*	Mean age (years)	Follow-up (months/mean)	Gender (% female)	Risser grade (median)	Risser grade (mean)	Sanders score (mean)	Sanders score (median)	Premenarche (%)
Samdani et al. [25]	USA	Retrospective	2014	IV	M	11	12.3	24	73	NR	0.6	NR	3.4	NR
Boudissa et al. [24]	France	Prospective	2017	IV	M	4	11.2	24	83	0	0.2	NR	NR	100
Newton et al. [26]	USA	Retrospective	2018	IV	L	14	11.3	28.9	65	0	0	2.4	2	100
Wong et al. [35]	Singapore	Prospective	2019	IV	M	5	11.7	49.2	100	0	0	2.6	2	80
Alanay et al. [42]	Turkey	Retrospective	2020	III	L	31	12.1	27.1	94	0	NR	NR	3	66
Hoernschemeyer et al. [40]	USA	Retrospective	2020	IV	L	29	12.7	38.4	90	2	1.9	4.3	4	NR
Miyanji et al. [28]	Canada	Retrospective	2020	NR	L	57	12.7	40.4	95	NR	0.05	3.3	NR	78
Newton et al. [36]	USA	Retrospective	2020	III	L	23	12	40.8	69	0	0.1	2.5	3	NR
Pehlivanoglu et al. [41]	Turkey	Prospective	2020	IV	L	21	11.1	27.4	71	NR	0.42	3.1	NR	0
Abdullah et al. [29]	Canada	Retrospective	2021	III	L	120	12.6	24	89	1	0.9	NR	NR	NR
Baker et al. [30]	USA	Retrospective	2021	IV	L	13	12.5	33	69	NR	0.8	2.9	NR	86
Baroncini et al. [43]	Germany	Retrospective	2021	III	L	45	13.2	24	84	1	0	5	5	NR
Hoernschemeyer et al. [33]	USA	Prospective	2021	NR	M	7	NR	24	NR	NR	NR	4.4	5	NR
Miyanji et al. [44]	Canada	Retrospective	2021	III	L	50	11.9	24	92	0	0.5	NR	NR	NR
Rushton et al. [37]	Canada	Retrospective	2021	III	L	104	12.7	37	92	0	0.5	3.4	NR	73
Samdani et al. [31]	USA	Prospective	2021	IV	L	57	12.4	55.2	86	0	0.47	3	3	85
Yucekel et al. [38]	Turkey	Prospective	2021	II	L	25	12.2	38.6	92	0	0	3	3	70
Bernard et al. [39]	England	Retrospective	2022	IV	M	8	12.6	64.5	100	NR	0.63	NR	NR	NR
McDonald et al. [32]	USA	Retrospective	2022	IV	M	51	12.3	24	80	0	0.4	3.1	3	NR
Averages						35.4	12.2	34.1	84.8	0	0.4	3.3	3	73.8

### Study cohort characteristics

Mean age was 12.2 years, mean follow-up was 34.1 months, 84.8% were female patients, and 74% were premenarchal. Table 1 displays pre-operative patient demographics. Appendix 3 displays the final follow-up demographics. All studies stated the pre-operative and a minimum 2-year post-operative Cobb angle. The median Risser score was 0, and the median Sanders was 3.

### Results of individual studies

**Fig. 2:**
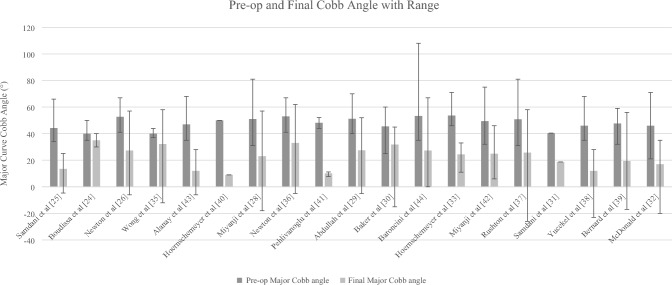
Graph of Cobb angle change

Initial mean major curve Cobb angle was 47.8° (CI 95% 42.9–52.7°) and decreased to 22.2° (CI 95% 19.9–24.5°) at minimum 2 years of post-operative. The mean main thoracic pre-operative major curve Cobb angle range was 31–81°, and at final follow-up, the range was − 26–62°. This indicates a mean reduction in major thoracic curve of 54% at the latest follow-up. The major curve Cobb angle results are displayed in Table 2 and Fig. 2.

**Table 2:** Clinical outcomes

Author	Pre-op major Cobb angle (°)	Pre-op Cobb range (°)	SD	Post-op major cobb angle (°)	Post-op range (°)	SD
Samdani et al. [25]	44.2	34–66	9	13.5	− 4.7 to 25.1	11.6
Boudissa et al. [24]	40.0	35–50	7.11	35	30 to 40	4.1
Newton et al. [26]	52.7	41–67	9.4	27.4	− 6 to 57	18.3
Wong et al. [35]	40.1	37.2–44	NR	32.2	− 12 to 58	NR
Alanay et al. [42]	47.0	35–68	7.6	11.8	− 6 to 28	12.1
Hoernschemeyer et al. [40]	50.0	NR	7	9	NR	17
Miyanji et al. [28]	51.0	31–81	10.9	23	− 18 to 57	15.4
Newton et al. [36]	53.0	41–67	8	33	− 5 to 62	18
Pehlivanoglu et al. [41]	48.2	44–52.1	NR	10.1	7.7 to 11.2	NR
Abdullah et al. [29]	51.2	40–70	7.8	27.5	− 5 to 52	11.6
Baker et al. [30]	45.5	25–60	10.6	31.8	15 to 45	8.7
Baroncini et al. [43]	53.3	35–108	13.3	27.3	0 to 67	14.4
Hoernschemeyer et al. [33]	53.6	46–71	NR	24.4	11 to 33	NR
Miyanji et al. [44]	49.4	32–75	8.5	24.9	6 to 46	9.5
Rushton et al. [37]	50.2	31–81	10.2	26.8	− 26 to 58	15.3
Samdani et al. [31]	40.4	NR	6.8	18.7	NR	13.4
Yucekel et al. [38]	46.0	35–68	7.7	12	− 23 to 28	11.5
Bernard et al. [39]	46.0	32–59	9.0	17	− 17 to 56	12.4
McDonald et al. [32]	46.0	21–71	11	17	− 20 to 35	11

### Summary statistics

**Fig. 3:**
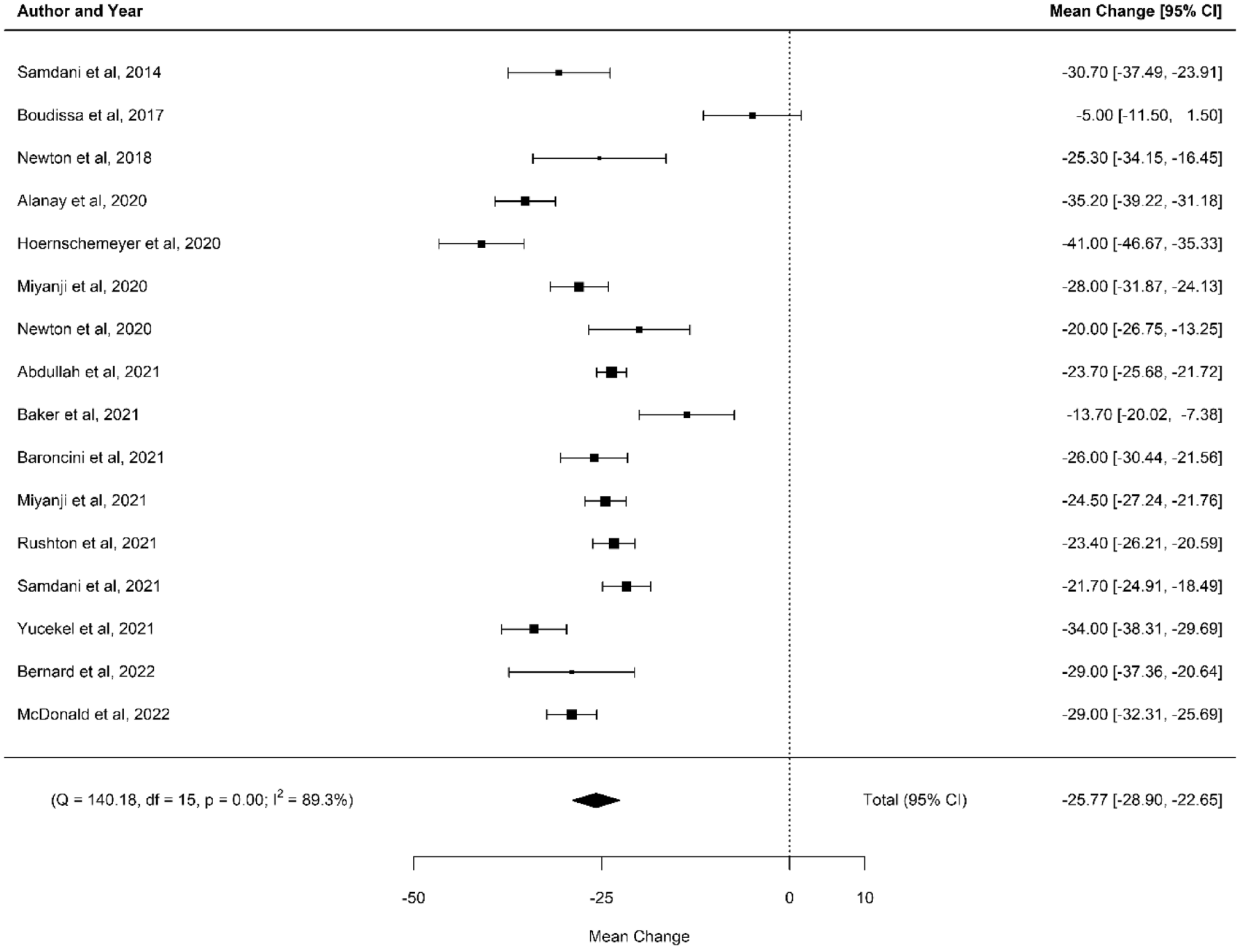
Forest plot

16 studies had sufficient data for a meta-analysis as displayed in Fig. 3. The mean difference in major curve Cobb angle was − 25.77° (CI95% − 28.9–22.65) with statistical significance (*p* < 0.01). *I*^2^ value of 89% and a significant *Q*-test for heterogeneity (*Q* = 140.18, df = 15, *p* < 0.01) indicate substantial heterogeneity.

**Fig. 4:**
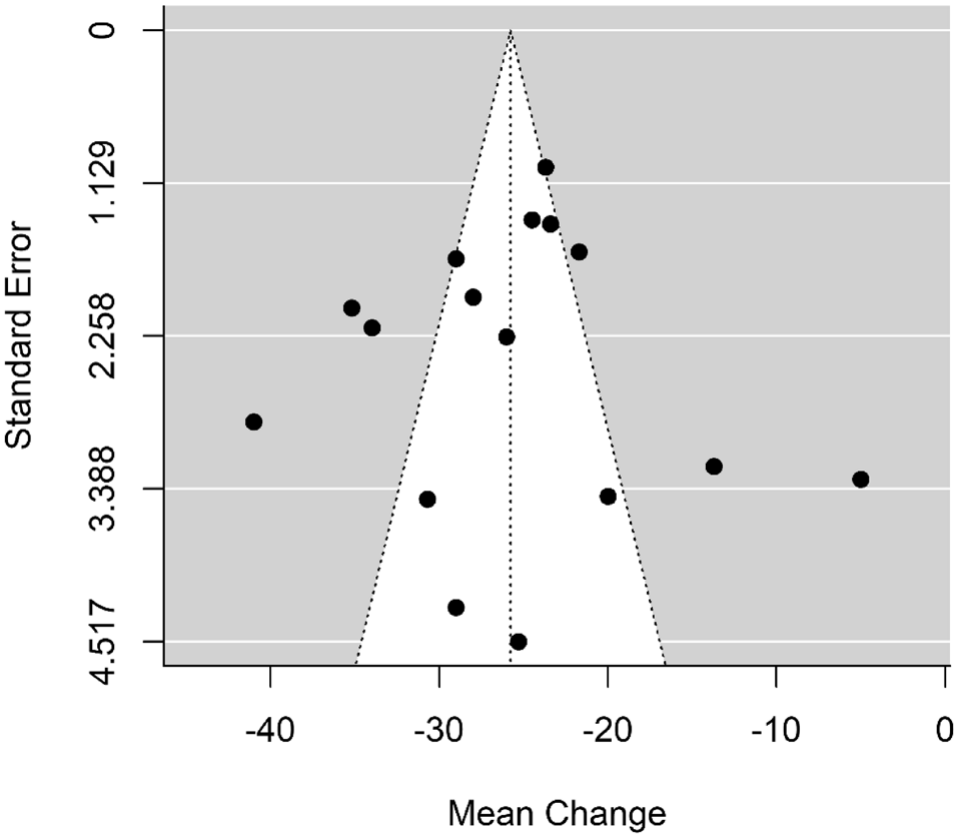
Funnel plot to assess for publication bias

Only one study [24] crossed the line of null effect, represented by the vertical line at 0 on the Forest Plot. This study had a small sample size with insufficient power, and an overall moderate risk of bias. There was no significant publication bias detected, as seen in Fig. 4.

### Surgical details

Most studies used a thoracoscopic approach and the Zimmer Biomet tether. The mean number of vertebral levels instrumented was 7.6, the mean surgical time was 223 min, mean intra-operative blood loss was 144 mL and the mean hospital inpatient stay was 4.9 days.

### Complications

17 studies reported complications, as seen in Table 3. The overall rate of complications was 23% (CI 95% 14.4–31.6%). The mean rate of tether breakage was 21.9% (CI 95% 10.6–33.1%), overcorrection was 11.4% (CI 95% 5.7–17.2%), re-operation was 11.4% (CI 95% 6.2–16.7%), spinal fusion rate was 7.2% (CI 95% 2.3–12.1%) and post-operative pulmonary complications was 6.7% (CI 95% 4–9.5%). Re-operations were most often removal of tether due to overcorrection, or replacement of a broken tether [25–31]. Complication rate was not reported in 2 studies [32, 33].

**Table 3:** Complication numbers and rates

Author	*n*	Breakage		Overcorrection		Revision		Fusion		Pulmonary		Overall	
		No.	%	No.	%	No.	%	No.	%	No.	%	No.	%
Samdani et al. [25]	11	0	0	2	18	2	18	0	0	1	9	5	27
Boudissa et al. [24]	6	0	0	0	0	0	0	0	0	0	0	0	0
Newton et al. [26]	17	8	47	4	24	7	41	4	24	2	12	25	70
Wong et al. [35]	5	0	0	2	40	0	0	2	40	1	20	5	60
Alanay et al. [42]	31	1	3	6	20	2	6.5	0	0	4	13	13	32
Hoernschemeyer et al. [40]	29	14	48	2	7	6	21	2	7	1	3	25	24
Miyanji et al. [28]	57	24	42	1	2	9	16	5	9	4	7	43	28
Newton et al. [36]	23	12	52	3	13	7	30	3	13	1	4	26	34
Pehlivanoglu et al. [41]	21	1	5	0	0	1	5	0	0	1	5	3	10
Abdullah et al. [29]	120	4	3	2	1.5	7	6	2	1.5	4	3	19	16
Baker et al. [30]	13	8	62	0	0	1	8	0	0	NR	NR	9	8
Baroncini et al. [43]	86	0	0	0	0	5	6	0	0	5	6	10	12
Hoernschemeyer et al. [33]	7	NR	NR	NR	NR	NR	NR	NR	NR	NR	NR	0	NR
Miyanji et al. [44]	50	NR	NR	1	2	0	0	1	2	NR	NR	2	4
Rushton et al. [37]	112	36	32	5	4	8	7	7	6	5	4	61	17
Samdani et al. [31]	57	NR	NR	5	9	8	14	1	2	NR	NR	14	14
Yucekel et al. [38]	25	5	24	6	24	4	16	2	8	2	8	19	24
Bernard et al. [39]	10	1	10	3	30	0	0	1	10	0	0	5	10
McDonald et al. [32]	51	NR	NR	NR	NR	NR	NR	NR	NR	NR	NR	0	NR
	Overall	7.6	21.9	2.5	11.4	3.9	11.4	1.8	7.2	2.2	6.7	14.9	23.0

## Discussion

### Major curve Cobb angle reduction

All studies demonstrated a reduction in the mean major curve Cobb angle at a minimum 2 years of follow-up compared to the pre-operative angle. Scoliosis is a progressive condition and during the pubertal growth spurt, the curve is expected to increase [34]. The mean difference effect showed an overall reduction of 26° of the major curve Cobb angle, with clear statistical significance (*p* < 0.01). A decrease of this size is significant because it indicates that the major curve has been prevented from progressing, as well as the overall deformity reduction.

11 studies had patients with a major curve Cobb angle reduction to a negative value, indicating overcorrection [25–29, 32, 35–39]. Negative angles are likely to skew the mean result, causing the mean difference due to VBT surgery to be larger than what is truly accurate. Without the data detail to separate those patients, it is impossible to say to what extent the overcorrection skewed the final mean result given.

Newton et al. defined VBT success as a final thoracic major curve magnitude of < 35° and no spinal fusion indicated [26]. 9 papers used this definition or similar when defining success [28, 30–32, 35–37, 39, 40]. This meta-analysis has shown the mean main thoracic Cobb angle to be 22.2° 2 years postoperatively, which can be considered clinically successful as a PSIF is not indicated. Thoracic curves in AIS tend to rapidly progress during the first 2 years of puberty [34]. The minimum 2-year follow-up time point was chosen in the hope that these patients would have completed their rapid growth spurt and be nearing skeletal maturity. If these patients were skeletally mature, then a Cobb angle of 22° would not be expected to progress. However, there was limited evidence in the skeletal maturity status of these patients at the final follow-up. Whilst Yucekul et al. found that the median Sanders score was 7 and the median Risser grade was 5 [38], Newton et al. found that 47% of the cohort remained at Risser 0–1 at final follow-up [26]. It would be advantageous that studies have a longer follow-up, until confirmed completion of growth, to definitively state whether VBT had been successful at avoiding spinal fusion.

### Negative surgical outcomes

The overall complication rate of 23% includes the most reported complications of tether breakages, overcorrection, revision, fusion and pulmonary complications. Tether breakage rate was 21.9%. As the tether is radiolucent, a break can be suspected if the angle between two adjacent screws increases by ≥ 5° between two time points [26]. The effect of a tether breakage has not been quantified, but several studies demonstrate some patients have progression of the curve after a breakage [26, 28, 36, 40, 41]. The clinical significance of the relatively high rate of tether breakages in this review is unknown and further research investigating the clinical outcomes after a breakage is required.

Whilst 21.9% of patients had a broken tether, 11.4% of patients experienced overcorrection, likely due to a greater growth potential at the time of surgery. These patients may have an undesirable outcome and a substantial deformity in the opposite direction. Further investigation correlating the demographics and surgical details between those with a broken tether and those who overcorrected may be useful to understand why these opposite consequences occur. As VBT is an emerging procedure, longer follow-up is required to see if the complication rate can be attributed to the surgical learning curve.

All patients had significant curves pre-operatively and the vast majority were destined towards a spinal fusion. After VBT, the reported rate of spinal fusion was 7%, indicating that the remaining 93% of patients were able to avoid a fusion at the minimum 2-year follow-up assessment. However, longer follow-up is essential to appreciate the true rate. Further correlation between patients that progressed and the timing of fusion would be beneficial, as slowing curve progression with VBT prior to PSIF may afford these young patients extra spinal height and avoid a more complex surgery.

There are currently two published systematic reviews on VBT [42, 43]. These papers used different inclusion and exclusion criteria to the current study. The study by Rialto et al. had a minimum follow-up period of 1 year [42], whereas the current study used 2 years as minimum follow-up to increase the likelihood of capturing changes seen within these growing patients. Bizzoca et al. included patients with lumbar only tethers [43], these patients were excluded in the current study as our aim was to directly compare thoracic VBT. Both of these systematic reviews did not include a meta-analysis, as the current study has done.

### Limitations

This review is proposed as an overview of the current state of VBT although it contains several limitations that require further research. One limitation is the reporting of the final major curve Cobb angles, as some studies have excluded those patients who had curve progression and required a PSIF [32, 44]. It is unclear in the remainder of the studies how and if these patients were reported. As the SD of the mean difference in major curve Cobb angle was not given in most papers, this number was estimated using the Cochrane formula. Further research with published SD may be more accurate for meta-analysis in the future.

14 studies have a negative major curve Cobb range due to overcorrection and the effect this has on the true mean result is unknown. Further analysis separating negative from positive major curve Cobb angles would be beneficial. This review contains majority retrospective pre–post studies and no randomised controlled trials (RCTs) as none have yet been conducted and published. Further research by method of RCTs would be more accurate at examining the effect of VBTs than from pre–post studies alone.

9 studies have a subsection of patients who do not meet the selection criteria are excluded (appendix 1). However, 3 papers include patients as the data was unable to be separated—2 patients with an additional lumbar tether [28], 3 patients with concurrent lumbar stapling [25], and 10 patients who had not reached the 2-year follow-up [27]. As the additional information has not been retrieved, it is not possible to separate these patients from the data set. This review has endeavoured to present very narrow selection criteria and multiple authors have generously supplied additional details. These 15 patients represent 0.02% of the data presented, so it was deemed warranted to include them in the final analysis, despite their limitations.

Another limitation is the lack of a control group. As the inclusion and exclusion criteria for VBT surgery are for a specific subset of patients, there is no equivalent surgery currently being performed that would be suitable for objective comparison. PSIF has been proposed as a comparison for VBT [45]. However, we maintain VBT is beneficial for a different cohort of AIS patients than PSIF. VBT is indicated for young patients that have large curves, not suitable for bracing, yet remain too skeletally immature for fusion surgery. These patients have a spinal deformity that will continue to progress requiring complex fusion surgery by the time their growth rate has slowed to a point whereby PSIF is permissible. Complete replacement of PSIF with VBT is not expected, but rather VBT exists as an option for certain patients who meet the strict criteria for VBT. Therefore, we conclude it would not be appropriate to compare PSIF with VBT surgery directly as the patient demographics for the two types of operation are very different.

The authors acknowledge the multiple biases that can be inherent in a purely bibliographic review. However, a carefully performed systematic review and meta-analysis that strictly follows the PRISMA guidelines can provide important insights into the effectiveness and safety of this procedure in the population for which it is indicated. Attempted mitigation of potential biases has been performed through our comprehensive search strategy, and our strict inclusion criteria.

### Implications for future practice

The results of this study demonstrate that VBT is effective at reducing and holding the scoliotic curve until 2 years of post-operative. Due to the nature of growing adolescents, studies examining the curves with longer follow-up are essential to determine the longitudinal effectiveness of this procedure into skeletal maturity. As the clinical effect from broken tethers has not yet been quantified, further studies that evaluate the change in major curve Cobb angle after a broken tether would also be beneficial. This will determine if a breakage is a complication resulting in continued curve progression, or if perhaps a tether breakage could be advantageous by contributing to the prevention of overcorrection. Further research into the timing of the procedure is necessary within a child’s growth, so that optimal reduction can be seen without overcorrection.

Further research is required to confirm the long-term effects of VBT, but with current knowledge, VBT remains an effective method to reduce the curve and prevent PSIF in skeletally immature patients with AIS.

### Supplementary Information

Below is the link to the electronic supplementary material.

Supplementary file1 (DOCX 16 KB)

Supplementary file2 (DOCX 21 KB)

Supplementary file3 (DOCX 19 KB)

## Data Availability

This study was registered with the Open Science Framework (URL https://osf.io/kx987/) and the review protocol can be accessed there. There were nil amendments to the registered protocol.
