# Supplementary material for: Vertebral body tethering for idiopathic scoliosis: a systematic review and meta-analysis
Source: Spine Deform. 2023 Jul 11;11(6):1297–307. doi: 10.1007/s43390-023-00723-9 (PMC10587225; doi:10.1007/s43390-023-00723-9)
Supplement: Supplementary file 1 — Supplementary file1 (DOCX 16 KB) [file 43390_2023_723_MOESM1_ESM.docx]

| Author | Excluded subset of patients |
| --- | --- |
| Samdani et al [25] | 3 patients had same day lumbar stapling, only 8 patients had VBT exclusively. I have unsuccessfully attempted to clarify the data with the author, the data includes those with lumbar stapling. |
| Boudissa et al [24] | 6 patients are included in this paper but only 4 have reached 2 year follow up. All 6 patients are included in demographics. I have excluded the two patients who did not reach 2 years follow up from the pre-op and final Cobb angle data. |
| Newton et al [26] | Of the 17 patients in this study, 14 have idiopathic scoliosis, and 3 patients have an underlying syndromic cause (Marfan’s, Prada-Willi, and triomy 4p and monosomy 13q). The demographics and results have been presented for the 14 idiopathic patients only when available. The percent female and the surgical details include all 17 patients. |
| Alanay et al [43] | Alanay had some patients at only 12 months follow up. 67.7% 21p had 2+ years follow up, 12.9% 4p had 18months, and 6 patients 19.4% had 12 months. Have emailed to clarify, currently met with “too hard to separate”. |
| Hoernschemeyer et al [40] | This paper had 31 patients, of these only 10 had a main thoracic curve treated with a single tether. Only these 10 patients are included in the Cobb angle analysis. However, the demographics included all 31 patients as that was not differentiated. |
| Miyanji et al [28] | Another study with 57 patients included a lumbar tether in 2 patients. I have unsuccessfully attempted to clarify the data with the author, the data includes 2 lumbar tethers. |
| Newton et al [36] | This article included a comparison group of patients who had had a posterior spinal fusion, this subset of patients was not included. |
| Baker et al [30] | Total n =17, 4 patients had a lumbar tether and have been excluded. 13 single thoracic tether only have been included |
| Baroncini et al [44] | One study with 86 total patients performed unilateral thoracic VBT in 45 patients. The author was contacted and supplied additional data to gain the demographics and results for the 45 thoracic patients, this data was included (not the total 86). |
| Hoernschemeyer et al [33] | The study by Hoernschemeyer (2021) included two patients with an additional lumbar tether. The results from these two patients were excluded in this study, there were minimal reported demographics and as such no major concern for bias. |
| Rushton et al [37] | 104 of the 112 patients are thoracic only tethers. The author was contacted and Cobb angle data for these 104 patients were included. The general demographics are for all 112 patients. |
| Bernard et al [39] | One study used VBT as growth modulation in 10 skeletally immature patients but also as an anterior scoliosis correction in 10 skeletally mature patients. For this review, only the patients who underwent growth modulation have been included. Eight of the growth modulation patients had a single thoracic tether only, so the remaining 2 patients were excluded from the Cobb data. |

Appendix 1. Studies with a subsection of excluded patients, or patients who were unable to be excluded but do not fit the selection criteria
