# Supplementary material for: Vertebral body tethering for idiopathic scoliosis: a systematic review and meta-analysis
Source: Spine Deform. 2023 Jul 11;11(6):1297–307. doi: 10.1007/s43390-023-00723-9 (PMC10587225; doi:10.1007/s43390-023-00723-9)
Supplement: Supplementary file 2 — Supplementary file2 (DOCX 21 KB) [file 43390_2023_723_MOESM2_ESM.docx]

|  | Quality Assessment Tool for Before-After (Pre-Post) Studies with No Control Group | | | | | | | | | | | | |
| --- | --- | --- | --- | --- | --- | --- | --- | --- | --- | --- | --- | --- | --- |
|  | Question number | | | | | | | | | | | | |
| Author | 1 | 2 | 3 | 4 | 5 | 6 | 7 | 8 | 9 | 10 | 11 | 12 | Total |
| Samdani et al [25] | Y | Y | Y | Y | Y | Y | Y | N | N | Y | Y | NA | M |
| Boudissa et al [24] | Y | Y | Y | Y | N | Y | Y | N | N | Y | Y | NA | M |
| Newton et al [26] | Y | Y | Y* | Y | Y | Y | Y | N | Y | Y | Y | NA | L |
| Wong et al [35] | Y | Y | Y | Y | NR | Y | Y | N | Y | N | Y | NA | M |
| Alanay et al [43] | Y | Y | Y | Y | Y | Y | Y | N | Y | Y | Y | NA | L |
| Hoernschemeyer et al [40] | Y | Y | Y | Y | Y | Y | Y | N | Y | Y | Y | NA | L |
| Miyanji et al [28] | Y | Y | Y | Y | Y | Y | Y | N | Y | Y | Y | NA | L |
| Newton et al [36] | Y | Y | Y | Y | Y | Y | Y | N | Y | Y | Y | NA | L |
| Pehlivanoglu et al [41] | Y | Y | Y | Y | NR | Y | Y | N | Y | Y | Y | NA | L |
| Abdullah et al [29] | Y | Y | Y | Y | Y | Y | Y | N | Y | N | Y | NA | L |
| Baker et al [30] | Y | Y | Y | Y | NR | Y | Y | N | Y | Y | Y | NA | L |
| Baroncini et al [44] | Y | Y | Y | Y | Y | Y | Y | N | Y | Y | Y | NA | L |
| Hoernschemeyer et al [33] | Y | Y | Y | Y | NR | Y | Y | N | Y | N | Y | NA | M |
| Miyanji et al [42] | Y | Y | Y | Y | Y | Y | Y | N | Y | Y | Y | NA | L |
| Rushton et al [37] | Y | Y | Y | Y | Y | Y | Y | N | Y | Y | Y | NA | L |
| Samdani et al [31] | Y | Y | Y | Y | Y | Y | Y | N | Y | Y | Y | NA | L |
| Yucekel et al [38] | Y | Y | Y | Y | Y | Y | Y | N | Y | Y | Y | NA | L |
| Bernard et al [39] | Y | Y | Y | Y | NR | Y | Y | N | Y | N | Y | NA | M |
| McDonald et al [32] | Y | Y | Y | Y | Y | Y | N | N | Y | N,Y | Y | NA | M |

Appendix 2a. Quality Assessment Tool for Before-After (Pre-Post) Studies with No Control Group

Y = yes; N = no, NR = not reported. Scoring for overall risk of bias assessment is as follows: 0-2 N, low risk of bias (L), 3-8 N moderate risk of bias (M), 9-11 N high risk of bias (H). NA = not applicable as this was a patient-level outcome. For question No. 5 NR – unable to calculate required sample size due to missing data (standard deviation), counted as N for risk of bias.

Appendix 2b. Criteria for the Risk of Bias Assessment

|  | Criteria |
| --- | --- |
| 1 | Was the study question or objective clearly stated? |
| 2 | Were eligibility/selection criteria for the study population prespecified and clearly described? |
| 3 | Were the participants in the study representative of those who would be eligible for the test/service/intervention in the general or clinical population of interest? |
| 4 | Were all eligible participants that met the prespecified entry criteria enrolled? |
| 5 | Was the sample size sufficiently large to provide confidence in the findings?   - Required sample sizes calculated in R using power.t.test - Mean difference and calculated std dev of the mean difference - Significance level = 0.05 - Power = 0.8 - Two-sided test |
| 6 | Was the test/service/intervention clearly described and delivered consistently across the study population? |
| 7 | Were the outcome measures prespecified, clearly defined, valid, reliable, and assessed consistently across all study participants? |
| 8 | Were the people assessing the outcomes blinded to the participants' exposures/interventions? |
| 9 | Was the loss to follow-up after baseline 20% or less? Were those lost to follow-up accounted for in the analysis? |
| 10 | Did the statistical methods examine changes in outcome measures from before to after the intervention? Were statistical tests done that provided p values for the pre-to-post changes? |
| 11 | Were outcome measures of interest taken multiple times before the intervention and multiple times after the intervention (i.e., did they use an interrupted time-series design)? |
| 12 | If the intervention was conducted at a group level (e.g., a whole hospital, a community, etc.) did the statistical analysis take into account the use of individual-level data to determine effects at the group level? |
|  | Overall risk of bias assessment (low, moderate, high) |
