# Supplementary material for: Vertebral body tethering for idiopathic scoliosis: a systematic review and meta-analysis
Source: Spine Deform. 2023 Jul 11;11(6):1297–307. doi: 10.1007/s43390-023-00723-9 (PMC10587225; doi:10.1007/s43390-023-00723-9)
Supplement: Supplementary file 3 — Supplementary file3 (DOCX 19 KB) [file 43390_2023_723_MOESM3_ESM.docx]

Table VI Skeletal maturity reported at final follow up

| Author | Reported skeletal maturity at final follow up | Reported outcome at final follow up |
| --- | --- | --- |
| Samdani et al [25] | No | - |
| Boudissa et al [24] | No | - |
| Newton et al [26] | Yes | 47% at Risser score 0 or 1 |
| Wong et al [35] | No | - |
| Alanay et al [43] | Yes | 74.2% of patients at Sanders 7 or 8, and median Risser of 5. 93% were post-menarchal. |
| Hoernschemeyer et al [40] | No | - |
| Miyanji et al [28] | Yes | Mean Risser score 4.3 (SD 1.02, range 0-5) at most recent follow up. |
| Newton et al [36] | Yes | The majority of patients were Risser 4 or 5 |
| Pehlivanoglu et al [41] | No | - |
| Abdullah et al [29] | No | - |
| Baker et al [30] | Yes | 68% patients were modified Risser 4 or 5 |
| Baroncini et al [44] | No | - |
| Hoernschemeyer et al [33] | No | - |
| Miyanji et al [42] | No | - |
| Rushton et al [37] | Yes | 84% were Risser 4 or 5 |
| Samdani et al [31] | No | - |
| Yucekel et al [38] | Yes | Median Sanders score was 7 (5-8) and median Risser score was 5 (3-5). |
| Bernard et al [39] | No | - |
| McDonald et al [32] | No | - |
